# The prevalence of heterozygous F12 mutations in Chinese population and its relevance to incidents of thrombosis

**DOI:** 10.1186/s12881-018-0557-1

**Published:** 2018-03-27

**Authors:** Xi Wu, Qiulan Ding, Xuefeng Wang, Jing Dai, Wenman Wu

**Affiliations:** 1 Department of Laboratory Medicine, Ruijin Hospital, Shanghai Jiaotong University School of Medicine, No.197 Ruijin Second Road, Shanghai, 200025 China; 2 Faculty of Medical Laboratory Science, Ruijin Hospital, Shanghai Jiaotong University School of Medicine, No.197 Ruijin Second Road, Shanghai, 200025 China

**Keywords:** Factor XII, Mutation, Thromboembolism

## Abstract

**Background:**

The contribution of moderate coagulation factor XII (FXII) deficiency to development of thromboembolism is still undetermined. We have tried to show the relevance of FXII deficiency to incidences of venous thrombosis by exploring the prevalence of *F12* gene mutations in Chinese patients with thrombotic disorders.

**Methods:**

One hundred and six patients with venous thromboembolism (VTE) and 220 healthy controls were enrolled in study. The coding region and flanking sequences of *F12* gene were amplified and sequenced to identify genetic variances. Patients with *F12* mutations were also screened for other thrombotic risk factors.

**Results:**

Heterozygous *F12* gene mutations were identified in 6 individuals with VTE and 10 healthy controls. Q336X and R66W were found in two healthy individuals; D291E was identified in a patient with DVT; and A343P was a recurrent mutation with a prevalence of 4.7% (5/106) in patient group and 3.6%(8/220) in healthy control. The prevalence of heterozygous mutations between the two groups had no significant difference. The association of A343P mutations with VTE was weak with an OR of 1.31 (95% CI 0.42-4.11). No other thrombophilia risk factors screened were positive in patients harboring heterozygous *F12* mutations.

**Conclusions:**

There were conflicting theories about the relationship between FXII deficiency and thrombosis formation. Heterozygous *F12* mutation decreases the plasma FXII activity approximately by half and cause moderate FXII deficiency. Although multiple mutations were identified in both groups, the link between *F12* heterozygous mutation and development of thrombotic disorders is weak and further studies are warranted to clarify their relationship.

## Background

Factor XII (FXII) is a part of contact activation pathway of blood coagulation and lack of it will lead to prolongation of blood clotting time initiated with artificial negatively charged surfaces [1]; however, patients with congenital FXII deficiency did not experience increased bleeding diathesis and some even suffered from thrombotic disorders [2]. Thus the role of FXII plays in blood coagulation has been an intriguing question since its discovery in 1950s with conflicting results from multiple clinical and experimental studies [3, 4]. The huge variation of plasma FXII level among normal populations complicates efforts to find out the relationship between FXII activity and pathogenesis of thrombosis. Since factor XII coding gene (*F12*) mutations will inevitably lead to decrease of FXII activity and are definite causes for FXII deficiency [5], we tried to explore the FXII deficiency in thrombosis disorders by screening *F12* mutations instead of measuring FXII activity.

## Methods

### Patients

Patients seeking bleeding and thrombotic disorders consultation in Ruijin Hospital, Shanghai, China, from 2012 through 2015 were screened. Totally 106 patients, including 68 males and 38 females (average age of 39.5 years, range 13~ 79), with venous thromboembolism (VTE) occurred in an early age (≤45 Years old) or recurrent VTE (≥2 times) without obvious provoking conditions, such as immobilization, using oral contraceptive, pregnancy, etc., were enrolled for studies. Blood sample was acquired via venipuncture and treated with 0.03% sodium citrate and leukocyte and plasma were separated by centrifugation at 2000 g for 15 min. Plasma samples were frozen at − 80 °C till further analysis and genomic DNA was extracted from isolated leukocytes using QIAamp DNA mini kit (QIAGEN, Hilden, Germany) according to the manufacturer’s instructions. Blood samples from 220 adults, constituted with 133 males and 87 females with an average age of 36.6(range 18~ 70), without history of thrombotic or bleeding complications were taken as normal control in current study. The study was approved by ethic committee of Ruijin Hospital affiliated Shanghai Jiao tong University and informed consents were obtained from all subjects enrolled.

### Genetic analysis of the *F12* gene

All exons of *F12* gene and noncoding sequences on either side of the coding region were amplified by PCR and sequenced directly. The genetic variation was identified by aligning sequencing results against *F12* gene reference sequence (NG_007568.1) in NCBI database. DNA samples from 220 healthy controls were also screened for *F12* gene mutation following same protocol mentioned above. The prevalences of *F12* mutations in patients with thrombosis and normal controls were compared.

The established genetic risk factors for thrombosis, including Factor V leiden mutation and prothrombin G20210A mutation were also tested among patients carrying *F12* gene mutations using PCR-RFLP methods as previously described [6].

### Coagulation assays

Citrate anti-coagulated plasma was subjected to a panel of assays to assess functional status of blood coagulation systems of patients with *F12* gene mutations. The traditional coagulation tests, such as activated partial thromboplastin time (aPTT) and prothrombin time (PT), were performed as first-line screening tests.

The plasma FXII activities (FXII:C) of patients with *F12* gene mutations were measured by a one-stage clotting assay using FXII deficient plasma on an ACL-TOP automatic coagulometer (Instrumentation Laboratory, Bedford, MA). Other common thrombotic risk factors,such as deficiencies of antithrombin (AT), protein C (PC) and Free protein S (FPS), presence of antiphospholipid antibodies and increased homeocysteine (Hcy) level, were also screened. Briefly, activities of AT and PC were detected using chromogenic substrate methods (Instrumentation Laboratory, Bedford, MA). Free PS antigen level was measured with the ZYMUTEST Free protein S kit (Hyphen Biomed, Neuville-Sur-Oise, France). Lupus anticoagulant was analyzed by a diluted viper venom time (DVVT) assay (Instrumentation Laboratory, Bedford, MA). The anti-cardiolipin antibody (ACA) and anti-β2 glycoprotein I (anti-β2GPI) levels were detected by ELISA (Euroimmun, Lübeck, Germany). Hcy levels were measured using the AxSYM homocysteine kit (Abbott, Lake County, IL, USA).

### *F12* C46T variant detection

Common *F12* C46T variant was detected using polymerase chain reaction-restriction fragment length polymorphism (PCR-RFLP) method according to conditions described by Zito et al. [7]. The prevalences of genotype C/C, C/T and T/T were determined in both patients and healthy control group.

### Statistical analysis

The difference between the prevalences of F12 heterozygous mutations in VTE and healthy control groups were analyzed by Chi-square test. Logistic regression analysis was adopted to estimate associations between recurrent heterozygous F12 mutation and VTE risk. Odds ratios (ORs) and 95% confidence intervals (95% CI) were calculated from the logistic model. The statistical analyses were performed with SPSS 19.0 software (SPSS, Chicago, IL, USA).

## Results

### Identification of heterozygous FXII mutations in thrombophilia patients

Multiple heterozygous *F12* gene mutations were identified in both groups. Two variants (A343P and D291E) were identified in 6 individuals of the VTE group (6/106, 5.7%);three variants (A343P, Q336X and R66W) were found in 10 individuals of the healthy control group (10/220, 4.5%). The prevalence of heterozygous individuals had no significant difference between the two groups (*p* = 0.6625). Mutations D291E, Q336X and R66W were each only identified once in a single subject; while the A343P mutation was recurrently found in 5 unrelated patients (5/101, 4.7%) and 8 healthy controls (8/220, 3.6%). The association of A343P mutation and VTE risk was low with an OR of 1.31 (95% CI 0.42-4.11).

### Coagulation assays

Patients harboring *F12* mutations included 4 males and 2 females, aging from 23 through 58 years old, and none of them had concurrence of other established risk factor for VTE. The *F12* gene mutations only led to moderately decreased plasma FXII:C, ranging from 33.2 to 73% (Table 1).

**Table 1:** Summary of F12 mutations and clinical presentation of patients with venous thrombosis

Patient ID	gender	Age range	thrombotic events,times	F12 mutation	FXII:C (%)	PC:A (%)	FPS:Ag (%)	AT:A (%)	Hcy (μM)	APS-Ab
1	Female	30~ 39	DVT,2	A343P	33.2	98	86	115	3.4	ND
2	Female	30~ 39	DVT,1	A343P	44.8	110	90	101	7.2	ND
3	Male	50~ 59	DVT,2	A343P	53.3	84	79	98	4.9	ND
4	Male	40~ 49	CVST,1	A343P	51.8	122	101	93	9.0	ND
5	Male	30~ 39	DVT/PE,2	A343P	56.5	105	76	109	5.7	ND
6	Male	20~ 29	PE,1	D291E	72.9	92	80	110	4.1	ND
RR					50-150	70-140	60-130	85-120	6.3-15	

*FXII:C*, factor XII activity; *PC:A*, protein C activity; *FPS:Ag*, free protein S antigen; *AT:A*, antithrombin activity; *Hcy*, homocysteine; *APS-Ab*, Anti-phospholipid syndrome-associated antibodies; *DVT*, deep vein thrombosis; *CVST*, cerebral venous sinus thrombosis; *PE*, pulmonary embolism; *ND*, not detected; *RR*, reference range

### Prevalence of *F12* C46T variant in patients with VTE and healthy control

**Fig. 1:**
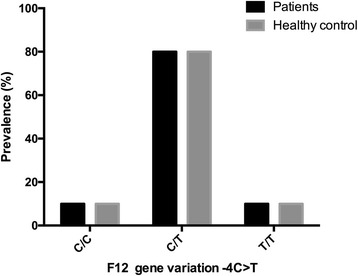
Prevalences of *F12* C46T variant in patients with venous thromboembolism and healthy control. The three genotypes C/C, C/T and TT have similar prevalences between patients with thrombotic complications and normal healthy control (C/C 56.4% vs 57.2%; C/T 41.5% vs 40.4%; T/T 2.1% vs 2.4%). The genotypes C/C and C/T are dominant in both groups

There is no significant difference of the prevalences of *F12* C46T variant genotypes between patients with thrombotic events and normal healthy control (*p* = 0.9633). Patients and healthy control group shared similar prevalences of three genotypes C/C (56.4%, 57.2%), C/T (41.5%, 40.4%)and TT (2.1%, 2.4%), with C/C and C/T dominating in both groups. (Fig. 1).

## Discussion

FXII is synthesized and secreted from liver in its zymogen form and is converted by autoactivation into its own enzymatically active form, FXIIa, on negatively charged surfaces, such as dextran sulfate, kaoline, etc [8]. FXIIa catalyzes prekallikrein (PK) and factor XI (FXI) to initiate the contact pathway of blood coagulation cascade; in the meantime, it helps breaking down of fibrin clot by converting plasminogen to plasmin and starting fibrinolysis [9]. FXIIa may also up-regulate directly or indirectly various mediators involved in the inflammation response [10].

FXII is encoded by *F12* gene on chromosome 5 and congenital FXII deficiency is a rare autosomal recessive disease with an estimated prevalence around 0.3% [11]. FXII deficiency will delay fibrin clot formation under artificial blood coagulation tests, however, it is mostly asymptomatic and almost no patients suffered from excessive bleeding. Counterintuitively, some even experience thrombosis.

Thrombosis model of mice lacking coagulation FXII showed that although FXII was not essential to physiological hemostasis, it might contribute to aggravation of thrombosis [12]. There were conflicting reports on FXII deficiency and thrombosis in epidemiological studies. Halbmayer WM et al. found that among 103 patients with recurrent venous thrombosis, 8% had reduced FXII level, suggesting that lack of FXII could lead to thromboembolism [13]. Another study performed in Caucasian also revealed higher prevalence of FXII deficiency in patients with DVT/PE (8.0%) than normal population (2.3%), supporting the notion that FXII deficiency might constitute a risk factor for thrombosis [14]. However, Koster, Ted et al. showed the prevalence of factor XII deficiency in patients suffering DVT/PE was not different from that of normal healthy control (6% vs 5%) [3].

The general population has a large range of plasma FXII level, with a normal range from around 80 to 120% [14, 15]. FXII level could be affected by multiple factors. There is racial difference [16], and oriental people might have lower FXII level than Caucasians; some SNPs within *F12* gene were also suggested to influence expression of FXII. Previous studies used different criteria for diagnosis of moderate FXII deficiency, with cutoff FXII level ranging 20~ 57% [3, 11, 14], which might confound the relationship between moderate plasma FXII deficiency and thrombosis.

Genetic polymorphism, such as *F12* C46T variant, was suggested to influence plasma FXII level [17], and it might be associated with decreased FXII activity and correlated with increased risk of thrombosis [18]. However, our result does not support this finding.

*F12* gene mutation impairs FXII expression and is the definitive cause for FXII deficiency. The homozygous *F12* mutation leads to complete deficiency of FXII. The heterozygous *F12* gene mutation reduces FXII level by half and the FXII:C level falls in a wide range between 20 and 60%, which overlaps with normal reference FXII level (50~ 150%). It could be difficult to distinguish between individuals bearing *F12* heterozygous mutations with healthy subjects who just happen to have a FXII level at lower end of normal reference range.

In current study, we screened *F12* gene of patients with unprovoked VTE and tried to evaluate the risk of thrombosis in those who had their FXII activity compromised because of *F12* mutation. Comparing with plasma FXII activity, *F12* gene variance identified is a more accurate indicator for moderate FXII deficiency. Our results showed similar prevalence of heterozygous *F12* mutations, including a hot spot mutation Ala343Pro, in thrombophilia patients and normal population. The FXII mutation Ala343Pro is characterized by inconsistent decrease of FXII clotting activity and antigen level in plasma. First identified in Japanese by Iijima, K.et [19], Ala343Pro has different prevalences among populations, and is overrepresented in East Asian races according to 1000 genome project. Animal studies suggested that FXII did not contribute to the physiological hemostasis, instead, it played a critical role in the pathological thrombosis formation. Significantly decreased FXII level (< 25%) could limit development of thrombosis in mice [20], however moderate FXII deficiency caused by heterologous *F12* mutation might not be able to protect thrombosis formation.

Anticoagulation therapy targeting FXII is both effective and safe, lessening risk of bleeding afflicted by most blood thinner used today, but sufficient suppression of FXII may be essential to the success of thrombosis prevention.

## Conclusions

Current study for the fist time reveals the prevalence of *F12* heterologous mutations in Chinese population and shows that there is no or only weak relationship between incidents of thrombosis and moderate FXII deficiency caused by heterozygous *F12* mutations.

## Abbreviations

ACA: Anti-cardiolipin antibody
anti-β2GPI: Anti-β2 glycoprotein I
APS-Ab: Anti-phospholipid syndrome-associated antibodies
aPTT: Activated partial thromboplastin time
AT:A: Antithrombin activity
CVST: Cerebral venous sinus thrombosis
DVT: Deep vein thrombosis
DVVT: Diluted viper venom time;
FPS:Ag: Free protein S antigen
FXI: Coagulation factor XI
FXII: Coagulation factor XII
FXII:C: Factor XII activity
Hcy: Homocysteine
ORs: Odds ratios
PC:A: Protein C activity
PCR-RFLP: Polymerase chain reaction-restriction fragment length polymorphism
PE: Pulmonary embolism
PK: Prekallikrein
PT: Prothrombin time
VTE: Venous thromboembolism
